# Extracts and Gleanings

**Published:** 1871-05

**Authors:** 


### PART II.

### Abridged Extracts and Gleanings from our Exchanges

*MULTUM IN PARVO.*

TUBAL PREGNANCY.—(*Spiegelberg : Arch fur Gynakologie.*)—The only authentic case of tubal pregnaucy, in which the fœtus attained maturity, is related by Saxtorph, and the history of this case is not sufficiently minute to be made use of; so that the full clinical report of a similar case, which occurred in the practice of Spiegelberg, is of great interest. The patient was a peasant woman, 44 years old, pregnant for the fourth time, and had reached the end of gestation without any abnormal symptoms, when fugitive labor-pains were soon followed by convulsions, coma, rapid prostration, and death in a few days. The urine was albuminous, and contained urinary casts. A *post mortem* examination disclosed a fully-developed dead fœtus, enclosed in membranes, and lying in a musculo-membranous sac which was formed by the distended and developed fallopian tube, the placenta having its attachment anteriorly. A minute microscopic report, by Waldeyer, confirmed the opinion that it was only the tube which had taken part in the development of the sac. Bundles of muscular tissue were found, the tissue of the ovary recognized microscopically, and, by careful manipulation, the folds of the right broad ligament were separated from one another up to the point where the sac commenced, which thus correspond with the position occupied by the fallopian tube, while a probe passed from the angle of the uterus into the sac along a short canal. Spiegelberg explains the fatal termination of the case by the eclampsia of the mother causing the death of the fœtus, subsequent separation of the placenta, hemorrhage into the sac, which was already distended to its utmost capacity, rupture, and death from peritonitis. The diagnosis was not made during life, owing to the normal course of the pregnancy, and the fact that the tumor corresponded so closely in position and size with the gravid uterus.—*Medical Times.*

DR. J. G. PINKHAM, in the *Boston Medical and Surgical Journal*, records a case of poisoning from forty drops of gelseminum sempervirens. By means of stimulants and friction, death was prevented.

CARBUNCLE.—Dr. J. C. Nott, of New York, having had his attention attracted by several articles in the medical journals, on the anæsthetic effects of carbolic acid when locally applied, tested the remedy in a case of carbuncle. All other remedies had proved unsatisfactory. An incision of about an inch and a quarter was made in the carbuncle, and stuffed with cotton saturated with pure carbolic acid. The whole surface of the hardened mass was also painted with the acid.

There was a sharp, burning sensation for a few minutes, when the pain subsided completely, and *he never complained of any afterwards.* Every day for a week the doctor continued to insert the acid in the same way into the cut, which sloughed all around to the depth of one-eighth of an inch ; the surrounding inflammation and induration subsided rapidly, and in a week there was nothing left to treat but the small open wound made by the knife and acid. Three other small carbuncles commenced, an inch or two from the large one ; they were all treated by incision and acid, and they all aborted.—*N. Y. Medical Journal.*

THE INCUBATION PERIOD OF SYPHILLIS.—Mr. MacCormac, of Belfast, communicates to the "Medical Press and Circular" of July 13th, his observations on two cases of constitutional syphilis, in which he has confidence as regards dates. In one case, forty-two days elapsed from the last intercourse till the appearance of local infection ; in the other twenty-eight days. In both cases it was presumed that the source of contagion was a secondary syphilitic lesion, which, until lately, has been rejected as a possible mode of contamination. From inoculations practiced on healthy persons, he concludes that the period of incubation is longer according as the source of infection is farther removed from the initial stage of chancre ; and Diday asserts that syphilis communicated from a secondary lesion is milder than when derived from a chancre. Consequently, an attack of syphilis is severe in inverse ratio to the period of incubation. Dr. MacCormac concludes, from the existence of this state of latency, that true chancre is not the initial point of syphilis, but the primary symptom of constitutional infection. Hence, all attempts to eradicate the disease by local treatment of the sore (excision or cauterization,) are worse than useless. He also regards mercurials as unable to prevent the later syphilitic symptoms, though perhaps able to modify them. He therefore prefers to delay their administration, in order to determine the natural severity of the case, which may be so mild as not to require a resort to mercury at all.—*New Orleans Journal of Medicine.*

Belladonna in the Treatment of Typhoid Fever.—Dr. B. Kelly, of Dublin, recommends very highly the use of belladonna in treating enteric fever. He does not prescribe it until the prominent symptoms are fully developed. If the patient be an adult, his dose is from twenty to twenty-five drops of the officinal tincture of the British Pharmacopœia every four hours, but must be varied to suit ages and constitutions. He says, "It completely changes the whole character and outward manifestation of the disease. Delirium, coma and subsultus quickly vanish, and are succeeded by calmness and clearness of intellect, by natural sleep, and complete control of all the voluntary muscles. Diarrhœa is checked, and healthy, consistent evacuations are established." He gives its most remarkable power as seen in the suddenness with which patients recover their intellectual faculties, and the full centrol of the muscles. In stopping its use it is necessary to do so gradually, to prevent serious consequences. All stimulants he interdicts, in every form and shape, while the patient is under the treatment of belladonna. Care must also be exercised that the patient does not get around too soon, which they are very much inclined to do while under its magical influence, and thus occasion a relapse.—*Med. Ind.*—*Chicago Med. Journal*, January 1871.

Dropsical Effusions.—Dr. S. P. Dutcher recommends the following prescription to remove dropsical effusions:

℞—Blue mass, . . . . gr. v.
Pulv. digitalis,
Pulv. squills,
Pulv. gamboge, aa, . . gr. x. m.

Make 10 pills. S. One pill every six hours.—*Med. and Surgical Reporter.*

Constant Vomiting and Eructations cured by Sulphurous Acid.—Dr. Chas. R. Dysdale, of the Metropolitan Free Hospital, London, reports (Lancet,) an interesting case, in which, after every available remedy was employed unsuccessfully for the relief of incessant vomiting and eructations, was at once relieved and permanently cured by half drachm doses of sulphurous acid, in one ounce of water three times daily.

Hypodermic Injection of Morphia in the Reduction of Hernia.—Dr. Ravoth reports (Berlin, Klin. Wochensch, June 7,) some remarkable results from the subcutaneous injection of morphia in cases of hernia which had previously resisted the taxis. Quarter grain doses were used.—*The Practitioner.*

CAUTERIZATION IN DIPHTHERIA.—In the 48*th Versammlung Deutsche Naturforscher und Aerzte*, Dr. Schuller stated that he had entirely abandoned cauterization of the pharynx, or conjunctiva in diphtheria. In numerous cases he had, as a crucial experiment, cauterized only one side of the fauces, and he had always been led to the same conclusioas :

1st. That the membrane remained attached longer on the side which he had cauterized than the other.

2d. That even the most energetic application of nitrate of silver failed to arrest the reproduction or to prevent the extension of the membrane.

3d. In some cases serious tumefaction and inflammation of the cervical lymphatics followed the application of the caustic.

In these views he was supported by Ebert, Stiebel, Cohen, Rinecker, and others, who direct the use of small pieces of ice to be constantly allowed to melt in the mouth, and employ a gargle of potass. chlor. alcohol, potass. permang., carbolic acid, etc.—*The Medical Times.*

IMPROVED FORMULA FOR CHALK MIXTURE.—

℞—Prepared chalk, . . . ℥ ss.
Glycerine, (best) . . . f. ℥ ss.
Pulv. g. arabic, . . . ʒ ii.
Cinnamon water,
Water, aa, f. ℥ iv.

Rub them together until they are thoroughly mixed. Paregoric, kino or catechu, may be added, if required. This mixture will keep during an entire summer, without fermenting. Glycerine may be nsed with great advantage, from this fact, to replace sugar in the food of children or adults, where there is enteric irritation or inflammation. Under these conditions, sugar almost always ferments. Pure glycerine does not ferment—is bland, and at the same time, becomes an excellent article of nourishment. Glycerine may be given infants when fed by hand, in place of sugar with food, whenever the bowels become deranged. Four to six drachms may be given daily. Only the purest article should be used.

BROMIDE OF POTASSIUM IN THE TREATMENT OF INTERMITTENT FEAER.—Repeated trials in Guy's Hospital have shown the very great value of the bromide of potassium, given in ague. The following formula is recommended :

℞—Bromide potassium. . . . ʒ v.

Tr. cinchonæ, yellow, . . ℥ iijss.

Spts. ammonia aromat. . . ℥ ss. m.

S. A teaspoonful in half a wine glass full of water, three times a day.

On Muriate of Ammonia as a Remedy for some Nervous Affections.—"Muriate of ammonia is one of those commonplace and unattractive substances which we, in this country, are little apt to credit with extensive remedial properties in disease." We quote the first sentence of an eminently suggestive paper by Dr. Anstie (The Practitioner, December, 1868), which treats principally of the employment of this remedy for the relief of (1) various kinds of pain, and (2) of certain cases of suspended secretion dependent on nervous exhaustion. Before very briefly describing some of the applications mentioned, we think it right to state that we are by no means prepared to coincide in Dr. Anstie's therapeutics, in so far as this is founded on physiological data. Under the first class the disease termed *myalgia* is said to be specially amenable to treatment by muriate of ammonia. Doses of from ten to twenty grains are recommended, and by their use this disease may be cured as certainly as ague by quinia. This class also includes various neuralgias proper, such as *migraine* (usually referred to disorders of digestion) and *clavus hystericus*, both of which Dr. Anstie believes to be distinet and primary neuralgias of the fifth cranial nerve. Of all the internal remedies that can be employed in these headaches, none is apparently so beneficial as the muriate of ammonia, its virtue depending on its mildly stimulant properties. It should be given in the same doses as for myalgia. In *intercostal* neuralgia, and especially in that form met with in suckling women or in phthisical patients, this remedy is also of great value, frequently "pain being relieved in half an hour." It may also be used with advantage in the milder forms of *sciatica*, which occur in young and debilitated persons; and in that somewhat obscure form of neuralgia termed *hepatic*. Among the therapeutic actions to relieve suspended secretion, Dr. Anstie expressos his canviction that muriate of ammonia "is the most powerful of all functional restoratives" of suspended bile secretion. He especially recommends it in those cases where the disease is produced by severe and exhausting mental excitement; and mentions that he has seen several instances in which two or three doses of twenty grains have caused a marked recommencement of biliary secretion. All these, and various other tharapeutical indications, are founded on the supposition that this remedy is a "pure tonic stimulant" to sensitive and to vaso-motor nerves. *Edin. Med. Journ.*

Lime-Water in the Treatment of Bright's Disease.—Kuchenmeister recommends in the treatment of Bright's disease, and of nephritis after scarlatina, the uses of large doses of lime-water, theoretically from its having the property of dissolving proteine. Lyon Medicale details the treatment, and says that caustic lime in solution, or any of the soluble salts of lime, will answer equally well. He has seen the urine increase from 30 grammes to 120 the first day, 180 the second, 300 the third, and up to 1020 the seventh day, under this influence; sometimes a slight hemorrage necessitates the disuse of this treatment; but the quantity of albumen in the urine sensibly diminishes.—*Med. Press and Circular.*

Cough Mixtures.—For the harassing and annoying coughs that frequently accompany many acute diseases, and arising from nervous irritation of the larynx, palate or other parts of the throat, an invaluable remedy is:

| | | |
|---|---|---|
| ℞—Sulph. morphia, | . . . | gr. i. |
| Dil. sulph. acid, | . . . | ʒ i. |
| Simple syrup, | . . . | ℥ ij. m. |

S. Half a teaspoonful to be given upon the tongue, and swallowed slowly. The persistent hackings of bronchial difficulties, and even of consumption, are often speedily relieved by it. An excellent cough mixture for constant use in the office or family, is this:

| | | |
|---|---|---|
| ℞—Syrup Tolu, | | |
| " Peru, | | |
| " Sanguinaria, | | |
| " Lobelia. aa, | . . . | ℥ i. |
| Tr. winter green, | . . . | ʒ i. m. |

S. Half a teaspoonful three or four times daily, or whenever indicated.

The following (Boston Medical and Surgical Journal,) formula is said to be of magical efficacy in all cases where an expectorant and sudorific are indicated; in catarrhal affections, spasmodic croup, pertussis, asthma, and in subduing mucus inflammation about the throat and air passages:

| | | |
|---|---|---|
| ℞—Tinct. lobelia, | . . . | ℥ ss. |
| " Blood root, | . . . | ℥ ij. |
| Oil Spearmint, | . . . | ʒ ss. |
| Empyreumatic syrup, | . . | ℥ v. m. |

S. Half a teaspoonful every two hours.

Morphia Collodian for Neuralgia.—Mix together one part of hydrochlorate of morphia and thirty parts of fexible collodian. Apply by means of a camel hair-pencil to the seat of neuralgic pains. Should these occur periodically, larger or smaller doses of the sulphate or the valerianate of quinia are to be given, besides.

Permanent Contraction of a Limb cured by Subcutaneous Injection of Atropia.—M. Desprez obtained a remarkable success in the case of a delicate young lady who had been for some years subject to articular rheumatism, and for a long time past had suffered from a fixed and extreme contraction of the arm, following in rheumatic inflammation of the shoulder-joint. When M. Desprez first saw her, there was no remaining swelling of the joint, but the limb was somewhat atrophied from want of movement. Frictions with belladonna accomplished some, but not very much good. A solution of sulphate of atropia was then prepared, 1 part in 400; and of this 25 drops (1-15 grain) were injected over the pectoralis major. Slight temporary intoxicative symptoms were produced. Three days later there was a marked improvement in the mobiiity of the joint; the muscles were less rigid, and there was less pain on attempting to move the arm. A second injection of thirty drops (1-13 grain) was made at the same point, and this was followed at intervals by three others, of 35 drops each (between 1-11 and 1-12 grain,) which completed the cure. The movement of the joint was quite restored, the atrophy has since disappeared, and the use of tonics and good feeding has subsequently much improved the patient's health.—*The Practitioner.*

To Make Hydrate Chloral Palatable.—The following combination serves to intensify the action of the chloral, and covers the acrid taste:

| | | |
|---|---|---|
| ℞—Hydrate chloral, | . . . | ʒ ss. |
| Chloroform water, | . . . | ʒ ii. |
| Syrp. orange or tolu, | . . . | ʒ iii. |
| Tinct. ginger, | . . . | gtt. vi. to xii. |
| Water, | . . . | ℥ iss. m. |

S. A part, or all at once.

The chloroform water is prepared by dissolving half a fluid ounce of chloroform in one gallon of water.

Rapid Cure for Buboes.—Dr. J. Grunfield, assistant to Sigmond, of Vienna, has had much success in extracting the pus by means of a hypodermic needle, India-rubber tube and syringe. Where the cavity fills again, a second operation of the same kind should be undertaken; and when the pus is unhealthy, weak solutions, either of carbolic acid or chlorate of potassa, should be injected and pumped out again by the same syringe.

Such patients as were so treated left the hospital much sooner than those whose buboes had been freely laid open,—*London Lancet.*

TURPENTINE IN UTERINE HEMORRHAGE.—Mr. Bradley, of Martley, near Worcester, a few years since, published some very valuable records of the utility of turpentine in hemorrhages of all kinds. As a restorative in certain cases of prostration, especially such as occasionally arise during the puerperal state, it is no less serviceable. Sometimes after a severe labor, accompanied or not with hemorrhage, great debility will ensue about the third day, characterized by a rapid pulse, tympanitic abdomen, and other symptoms not connected with peritoneal or other fever, yet threatening the advent of a typhoid condition. Here turpentine, both as an injection and by the mouth, is invaluable. Mr. Yarraway, of Faversham, records (British Med. Journ., July 10, 1869,) a case of this character occurriug in a primipara on the third day after labor. One ounce of turpentine, diffused in mucilage, was injected as high as possible into the rectum ; the patient had been previously insensible, with cold aud sweating skin, and commencing shrivelling of the surface, but in four or five minvtes after the turpentine injection, the respiration became freer, she soon opened her eyes, deglutition became possible ; after which nourishment was administered with the best effects.

ERGOTINE AFTER AMPUTATION.—At a meeting of the French Academy, on the 30th of November, Mr. Bonjean sent in a note to the effect that when ergotine has been given after operation, the mortality is thereby greatly diminished. Mr. Bonjean states that at the Hospital of St. Andre, in Bordeaux, the mortality after amputation, which had been three fourths, had been reduced for the last year to one-fifth. The surgeons at the hospital give the patient, immediately after the operation, and for a space of fifteen days, from 2 to 3 grammes (from 1.2 to 1.9 dwts. troy) in a draught. The chief remedial effect of this is to diminish or prevent suppuration.—*The Practitioner.*

AMBLYOPIA CURED BY HYPODERMIC INJECTION OF STRYCHNIA.—Dr. Jos. Talko, of Tiflis, reports (Klin. Monatsblatter f. Augenheildunde, Mai) a very interesting case of amblyopia cured entirely and solely by this method. The doses were 1-12 raised gradually to 1-4 of a grain of nitrate of strychnia ; the injection was made in the neighborhood of the affected eye ; it seemed to answer best when done in the supra-orbital region. The cure may be said to have occupied about seven weeks, and was then complete. It is remarkable that such large doses, repeated as often as once a week, produced neither local inconvenience nor constitutional poisoning, with the exception of the trivial symptoms.—*The Practitioner.*

Treatment of Herpes Zoster by Belladonna Ointment.—Dr. Dauvergne, *pere*, carefully discusses (Bulletin Gen. de Therapeut., Feb. 20, 1869,) the various means that have been recommended to assuage the sufferings of patients affected with this disease, and recommends, as the result of his experience, an ointment composed of five parts of extract of belladonna to thirty parts of axunge. He believes that this ointment has two advantages due to the belladonna it contains. In the first place, it favorably affects the local hyperæmia, in virtue of its well-recognized property of exciting the contractility of the minute blood-vessels; and besides it directly subdues and removes neuralgic affections.—*Edin. Med. Journ.*

Chorea cured by Ether spray applied to the Spine.—M. Mazade records (Lyon Medical, 4 Juliet) an interesting case in which severe chorea was effectually treated in the above manner. The patient was a young man, aged eighteen; the disease had lasted many months, and after a brief interruption (during an attack of varicella,) had returned with increased violence. Fifty grammes (14 drachms) of ether were applied, in spray, along the whole length of the spine with a Richardson's apparatus; the skin was slightly reddened, but not rendered anæsthetic. The next morning the patient announced that he felt much better, although the external symptoms were not very materially changed. The ether deuche was administered three times more, and a remarkable improvement was then observed; the treatment was therefore continued, and at the end of about eight days the patient could write legibly, whereas the affection of the hands had been so severe as to entirely prevent even his feeding himself. He was so well as to be about to leave the hospital, about three weeks after the commencement of the ether treatment, when he was attacked with typhoid fever. The latter affection proved slight, and was not followed by any return of the choreic symptoms.—*The Practitioner.*

Sulphurious Acid in Typhoid Fever.—Dr. Wilkes, of Ashford, Kent, affirms (*Med. Press and Circular*), that he has had very great success in the treatment of typhoid fever by means of small doses of sulphnrous acid. He gives the acid with syrup of oranges in water, in doses of from two and a half to twenty minims every four hours, until the patient complains of tasting, as it were, lucifer matches. He alleges that this remedy arrests the further development of the fever-poison, and thus exterminates the fever. "Briefly it is an antidote." Of 113 cases of typhoid fever treated, only two died, and these two did not take the acid.

Calabar Bean in Tetanus and Trismus Neonatorum.—Dr. Monti has been trying this treatment in four cases of tetanus in new-born children, and one case of traumatic origin in a boy four years old. The traumatic case and two of the infants recovered; the remedy was mostly administered by. subcutaneous injection. The author remarks on the extreme rarity of. favorable results in this disease, and concludes, from his own experience and that recorded by other observers, that calabar bean offers a much better prospect of positive therapeutic results than does any other drug. The proper dose for subcutaneous injection of an infant a few days old seems to be 1-30 to 1-60 grain of the extract; in the boy of four years old Monti injected as much as ½ grain, and a total of 6½ grains of the extrhct were given altogether, by stomach and by skin.—*The Practitioner.*

Cardiac Neuralgia.—Dr. Anstie read a paper at the late meeting of the British Medical Association at Oxford, in which he endeavored to prove that the essential pathology in a whole group of cases which exhibit more or less typically the features of what is known as angina pectoris, are *neurotic*, and that the very various cardiac and vascular lesions which are found in different cases are but accidents and complicatious. A system of treatment based on this principle proves greatly more successful than any other: the two remedies which have yielded him the best *permanent* results are arsenic given by the stomach or by inhalation, and strychnia subcutaneously injected in doses of from 1-20 to 1-40 grains. The benefit produced by these remedies is very remarkable. The best palliative for the actual attacks is ether, in teaspoonful doses.—[Practitioner.

Application for Cancer.—An Italian medical journal recommends the following application for cancer of the breast: Concentrated acetic acid, 15 parts; creosote 3 7-8 parts; water 450 parts. A case is mentioned, in which a cancer was removed, and cicatrization completed, in six weeks. The application was made on lint, four or five times daily.—*Dental Times.*

Hydrate of Chloral and Morphia in Tetanus.—Dr. Groneman has published the case of a man of forty-four, who suffered from tetanus in consequence of a shot in the chest. Chloral alone did not relieve the spasms, but when morphia was given with it, the improvement was manifest. The prescription was as follows: Hpdrate of chloral, 150 grains; hydrochlorate of morphia, 3 grains; distilled water and simple syrup, of each two ounces; the fourth part night and morning. The man recovered.—*American Journal of Dental Sciences.*

Things not Generally Known.—The Pharmaceutical Journal publishes a remarkable instance of unforseen danger arising from the facility with which oxide of silver is reduced by contact with vegetable extracts in common use. A medical man prescribed twenty-four pills, each containing two grains of the oxide of silver, a twenty-fourth of a grain of muriate of morphia, and a sufficiency of extract of gentian; the pills being coated with silver in the usual manner. The pills were delivered to the patient in an ordinary pill-box, but the lady, being in her nursery, and having no pocket in her dress, placed the box in her bosom, probably next the skin. In three-quarters of an hour a severe explosion occurred; her under-clothes were reduced to tinder, and her right breast was seriously burnt. The patient fortunately had presence of mind enough to seize the part with both hands, and thus extinguish the flame. We learn from Mr. Hills that a similar occurrence has been known in compounding the extract of colocynth with the oxide of silver, and that with creosote or oil of cloves this salt is reduced to the metallic state, with the production of heat often amounting to an explosion. In fact, many of the essential oils reduce the oxide of silver, and one of the processes for silvering glass is founded on the fact, oil of cloves being usually employed in the operation. We may mention that when glycerine and permanganate of potash come in contact, heat is evolved, sometimes resulting in flame. An instance has occurred in which a wound was covered with the glycerine of starch, and then sprinkled with powdered permanganate of potash, when the heat produced became unbearable.—*Lancet.*

Gleet and Leucorrhœa cured by the use of Bromide Potassium.—A. H. Kinneor, M. D., of Metamora, Illinois, reports cases of gleet und leucorrhœa cured by the use of bromide potassium. He prescribes it in doses of xx gr. twice a day, thirty minutes after meals. He thinks it beneficial in either the uterine or vaginal form of the latter disease. "We obtain, as it were, two effects from the use of the drug—alterative and nervo-sedative, producing a marked sedative effect upon the genital organs, allaying all irritation of the parts; in fact, putting them at rest, thus giving the medicine every chance to produce its alterative effect upon the parts diseased."—*Indiana Journal of Medicine.*

Possible Duration of Pregnancy.—In the course of an action for damages for the seduction of a young woman, the question of the possibly protracted duration of gestation was raised. The alleged father had had no access to the mother of the child later than 301 days before its birth, and he naturally disputed his liability. Dr. Tanner deposed that the ordinary period was 270 to 280 days, but might be exceeded by two, three, or even four weeks. He thought there was no inconsistency in the present case (from April 15th to February 9th—that is, 301 days). He had not known any case himself in which the ordinary period had been exceeded by a week, but he had no doubt there were such cases. He had heard of such. Mr. James F. Clark deposed that there were cases on record extending over more than 301 days. Sir James Simpson had recorded a case of 310 days. Dr. Barnes deposed that the ordinary period was 271 days. He had known cases of 280 and of 285 days. He thought it very improbable, but did not like to say it was impossible, for gestation to extend over 301 days. It was so improbable that he did not believe it. Dr. Tyler said that the longest period of excess he had known was a fortnight. Dr. Reid—a most accurate observer—had recorded forty-three eases of protraction, the longest of which was 300 days. Dr. Smith considered that case as reliable as any doubtful case could be. The verdict was for the plaintiff—damages £200.—*British Med. Journ.*, March 5th, 1870.

Speaking and Singing without a Tongue.—In the Transactions of the Philosophical Society, published between 1742 and 1744, there is an account of Margaret Cutter, who, when four years old, lost her entire tongue from a cancerous affection; but who, nevertheless, afterward retained the power of taste, swallowing, and speech, without any imperfection whatever. She not only spoke as fluently, and with as much correctness, as other people, but also sung to admiration, articulating with distinctness all her words while singing. What is not less singular, she could form no idea of the use of a tongue in other persons. This remarkable case was brought before the Royal Society, under certificates of attestation from the minister of the parish, a medical practitioner, and another respectable citizen, well known in Suffolk, where she resided. On account of the extraordinary character of the case, the Society requested an additional report upon the subject, and from another set of witnesses, named by the Society for the purpose, and for whom they drew up the necessary questions, and marked out the proper course of examination. The second report coincided with the first in all particulars, and shortly afterward, the young woman was brought to London, where she confirmed the account by personally appearing, and speaking and singing, in the presence of the members of the Royal Society, and many other persons.—*College Courant.*

Epistaxis.—*Messrs. Editors:* Several articles have, of late, appeared in your "Journal" on a mode of stopping hemorrhage from the nose. The following case presents another method, which may be in common use, but which I have not seen anywhere mentioned:

A month ago I was called to an Irishwoman, who had been bleeding from the nose, most of the night, as was said. I found her, with discolored eyes and flattened nose, still bleeding, though not profusely. In a vessel beside her bed was, apparently, more than a pint of arterial blood, besides quite a quantity spattered over the bed, the furniture and the floor. I threw her head back, somewhat, and poured, from a spoon, a little dilute Monsel's styptic into each nostril. The hemorrhage at once ceased, and did not recur.—" **," in *Boston Medical and Surgical Journal.*

Therapeutic Effects of Gelseminum.—Dr. E. A. Anderson, of North Carolina, experimented very extensively during the war with the gelseminum, in his efforts to find some agent that would serve the purpose of quinine in intermittent and remittent fevers. He now uses this agent—the gelseminum—almost exclusively in intermittents, especially in children, to whom it is especially adapted, from its absence of any of the unpleasant taste of quinine. He has seldom been disappointed in its effects. He employs the tincture (℥iv. bruised root to Oi. alcohol) or the fluid extract of Tilden. Dose in the proportion of twenty drops to an adult. It should, like quinine, be given before the expected paroxysm; four to six doses should be given before its onset is expected. He says: "I have found it a reliable agent in intermittent, remittent and typhoid fevers; acute and chronic rheumatism; in inflammations of the lungs, pleura and pneumonia. I have, for several years, used it almost exclusively in pneumonia, in place of veratrum, and consider it the best agent in this disease. You can reduce the force and frequency of the circulation with it, as certainly as by digitalis, venesection, or veratrum, without the distressing effects of these agents, while, at the same time, it allays the morbid heat, restlessness and irritating cough."—*Journal Materia Medica.*

Dr. Thomas Mead, in the "National Medical Journal," reports a case of strychnine poisoning successfully treated with hydrate of chloral. The patient, with the intention of suicide, took two grains of strychnine, and was shortly after thrown into violent tetanic convulsions. After an ineffectual attempt to produce emesis with ipecac, one drachm of hydrate chloral in solution was given, followed in five minutes by marked relief of the spasms. In an half hour the convulsions returned, and were again promptly controlled by a second dose of the chloral. For the next eight or ten hours there were threatened recurrences of spasm, in each instance quieted by chloroform inhalations. The patient made a good recovery.

PURPURA IN CHILDREN AND ITS TREATMENT BY SECALE CORNUTUM.—Dr. Bauer describes two forms of purpura; and states in the *Deutsche Klinik*, No. 35, he has had frequent opportunities of treating this affection in young patients. The most common form is the purpura simplex, which occurs in suckling infants and young children belonging to the rich as well as the poor classes of life. This affection is generally very slight, and seldom requires any special treatment. The second form, purpura hemorrhagica, which is much more severe, is characterized by hemorrhages from the nose, lungs, intestines, and womb. For this affection, which is generally very obstinate and resistent to remedies hitherto proposed, Dr. Bauer has administered, with marked and speedy results, ergot of rye, either as powder or in an infusion. The usual dose is from eight to ten grains, given, according to circumstances, once, twice, or thrice in the day. Under the influence of this drug, hemorrhage is arrested, and the effused blood absorbed in from one to three days, or at the very latest, by the sixth day. The use of ergot does not, however, guard against relapse.—*Brit. Med. Journal.*

TREATMENT OF ITCH, AS PURSUED IN PRIVATE PRACTICE—BY A. HARDY.—Mr. Hardy employs an ointment a little less irritating than that of Helmerich. It consists of sulph, sublimat., 2 parts; potass. sub. carb., 1 part; adipis., 12 parts; After a bath before going to bed, the patient is subjected to a general friction with this ointment, and to another on the following morning, without having taken any previous bath. In the evening the patient again takes a bath to wash off the ointment, and then is treated by emollients till the irritation caused by the disease and the frictions has subsided.—*Gazette des Hopiteaux.*

A USEFUL PRESCRIPTION—for the exhibition of the salts of morphia, in cases where they would, given singly, cause nausea and vomiting, is the following:

| | | |
|---|---|---|
| ℞—Morph. sulph., | . . . | gr. j. |
| Ext. aconite rad. alc., | . . | gr. ij. |
| Ext. belladonnæ, alc. | . . | gr. iij. |
| Ext. hyoscyamæ, | . . | gr. iv. M. |

Ft. pil. No. viij. Signa: one every half hour, till relief of pain.—*N. W. Med. and Surg. Jour.*

Camphor as an Application in Chancre.—M. Champouillon, a Surgeon-Major of the French Army, states (*Recueil de Med. Mil.*, February) that during the last eleven years he has dressed chancres, whether hard or soft, with finely powdered camphor, and has derived the greatest advantage from the practice. The chancre begins to clean at once, and an ordinary cicatrization is not uncommon at the end of ten or twelve days. When the ulcer is very large or phagadænic, or the constitution is bad, more time of course will be required; but even in these cases the aspect of the sore is rapidly changed for the better. Buboes are also of rarer occurrence. It succeeds best in the chancres covered with the prepuce, as the dressing is less disturbed.—*Med. Times and Gaz.*

Chloral Hydrate in Puerperal Mania and Puerperal Convulsions.—Dr. Phillips stated to the Obstetrical Society of London that during the past nine months he had used the hydrate of chloral extensively in the puerperal state, especially in 5 cases of puerperal mania, and two of puerperal convulsions. In four of the five cases of mania its action had been very beneficial, while in the fifth it failed to produce sleep, though given in full doses. In one case of mania the patient had no sleep for three days, though opium had been given, but within five minutes of taking half a drachm of hydrate of chloral she fell asleep for four hours, and again for five hours more. In another case, on the fourth day it was given in full doses, and the next day the patient was quite rational. The chloral hydrate was very suitable in the restless, sleepless condition not uncommon after delivery. A drachm dose produced no effect in one case of convulsions, while in another, in which the paroxysms were severe and frequently repeated, the action of the chloral was very marked. It was very satisfactory to receive such a favorable account of chloroform in obstetric practice from one so accustomed to its use as Dr. Kidd was. He (Dr. Phillips) had seen it used extensively in abnormal labors without untoward effects, and this notwithstanding that he had it used continuously for twelve consecutive hours in puerperal convulsions.—*Med. Times and Gazette.*

Permanganate of Potash in Gonorrhœa.—Dr. Thos. Narden commends this drug (*London Lancet*, Dec. 1870) as an injection (gr. v—xv to f ℥ i) in this disease. Great care should be taken to have dish and syringe clean.

Leeches and Mustard.—A correspondent in the *London Lancet* states that the application of mustard previous to the application of leeches causes them to take hold with great rapidity and avidity.

Value of the Preparations of Conium.—Dr. John Harley in an article on this subject in *The Practitioner* (Dec. 1870), states that the advantage of the *green fruit* over every other part of the plant is so clear and decided, that nothing need be said in favor of its selection as the basis of the tincture and extract. The extract of the Pharmacopœia is a scandal to the present state of medical knowledge, and a spirituous extract of the green fruit ought as soon as possible to take its place; then indeed we shall have an extract of which the proper dose will be "from two to six grains," instead of from 20 to 60 grains or more, which is the efficient dose of the present extract.

Kali-Kuki—A New Tonic.—Mr. M. C. Cooke, in a late number of the *Pharmaceutical Journal*, gives a description and an account of the properties of this plant, which has long been considered to be the black hellebore, but which really belongs, not to the Ranunculaceæ, but to the Serophulariaceæ. Though unknown in the English market, it is well known throughout India. The drug consists partly of the root and partly of the stem of the plant. The root part is very light and brittle, about the size of a goose-quill, brownish-white in color externally, and deep black internally, with short waxy fracture. It is said to be a very valuable tonic, Assistant Surgeon Mourdeen Sheriff considering it equal to gentian and columba, and superior to chirayta. As a dose, ten or twenty grains as a tonic, and from twenty to forty as an antiperiodic, are recommended.—*Lancet.*

*As a Remedy for Diphtheria*, Dr. Rothe, of Altenburg, recommends the following: Acidi carbolici, alcoholis aa 1-10, tinct. iodin. 0-5, aquæ dest. 5-0 m. The mixture is applied three times daily to the affected parts of the throat, and the removable membrane is detached with the same brush, so that the mixture touches the mucous membrane. The patient also uses a gargle of water, to a teacupful of which from 10 to 15 drops of the mixture is added. In severe cases the application is renewed every three or two hours, if possible, also during the night. Dr. Rothe relates 15 cases, of which but one terminated fatally, and in this the diagnosis "diptheritis" was doubtful.—*N. Jahrb. f. Pharm.*

Iodide of Potassium in Bright's Disease.—Prof. Cryni, of Brussels (*Brit. Med. Journ.*, *Wiener Med. Wochenschrift,*) strongly recommends this salt, in large doses, in the second stage of Bright's disease. Favorable results by this treatment are also said to have been obtained by Drs. Baudon and Semmala, of Naples, and Dr. Caspari, of Meiningen.

A Remedy for Asthma.—Dr. James S. Bailey, of Albany, says in the *Medical Record :* Having during the last fifteen years prescribed many remedies with indifferent and uncertain success, after much careful study I have prepared the following formulary, which has, during more than half this period, acted to my entire satisfaction. I now place it at the disposal of the profession, hoping it may meet with the same success in their hands as in mine, in relieving the distress of this unfortunate class of sufferers:

| | | |
|---|---|---|
| ℞—Syrup of tar compound, | . . | oz iv. |
| Sulphuric ether, | . . | oz. ii. |
| Pulv. gum acacia, | . . | drs. vi. M. |

Take one teaspoonful every two or three hours until relieved. Oftentimes two or three doses are quite sufficient to relieve an aggravated attack of spasmodic asthma.

As the syrup of tar compound is my own preparation and not pharmaceutical, it is necessary to give the formula for its preparation:—

| | | |
|---|---|---|
| ℞—Picis liquidæ, | . . . | oz. iv. |
| Scillæ acet. | . . . | O. j. |
| Antim. et potass. tart. | . . | grs. xvi. |
| Magnesiæ carb. | . . | oz. ss. |
| Sacch. albi, | . . | oz. xxx. |
| Spts. vini rectificati, aa, | . . | f. oz j. |

Mix the tar and carb. magnesiæ intimately in a mortar, adding the alcohol first and then the ether, then add the acet. scillæ and throw the whole upon a stout filter. Having added sufficient acet. scillæ (if may be) to make the filtered liquid measure O. j. proceed to make the syrup by the usual U. S. P. formulary, taking care to apply a gentle heat. Lastly, strain through flannel and add the tart. antimonii in solution.

After the paroxysm is relieved, the patient is left in a relaxed and debilitated condition; it is then necessary to institute a tonic course of treatment. A generous diet, with simple bitters or mild preparation of iron, is generally sufficient to restore the system to its accustomed health.

Sulphites in Pyæmia.—Dr. Wm. MacCormac (*British Med. Journal*) says, "Sometime ago I had frequent opportunities of trying Prof. Polli's antizymotic treatment by the bisulphites of soda or magnesia. It always appeared to me to do a good deal of harm, and never much if any good. Diarrhœa was induced by it, as well as vomiting, the abdomen swelled up with flatulence, and food was sooner refused."

Iodoform has lately been brought prominently to the notice of physicians in this country as a remedy for chronic ulcers (Proc. Penn. Med. Soc., 1868,) obstinate neuralgia, scrofula, strumous opthalmia, consumption, and even in cancer is stated to have relieved the excruciating pain of this malignant disease, without seeming to arrest the same (*Med. and Surg. Reporter*, Phil.; Vol. 16, 17, 19). It is also a valuable dressing in chancre.

It is best administered in pill form, one to two grains, three times a day. Quevenne's iron may often be advantageously added. Externally it is used as an ointment, one-half to one dram of iodoform to one ounce of lard, or it is dissolved in hot alcohol, and glycerin added: these to be used *pro re nata.—The Pharmacist.*

Chloræthyl, a new Anæsthetic.—(*Boston Jour. Chem.*)—"The new anæsthetic, *chloræthyl*, *æthyliden chlorid.* discovered by the distinguished Dr. Oscar Liebreich, of Berlin, the discoverer of chloral hydrate, is really an agent of great promise. We have during the past two months experimented with it considerably; and we find in our own case it produces anæsthesia quickly, and is free from any unpleasant after-symptoms. It certainly produces less nausea than chloroform or ether, the insensibility is very profound, and the agent has a pleasant odor. These are important considerations. The only drawback is its high cost, it being ten times greater than chloroform. With improved methods of manufacture, this objection may be overcome."

Oil of Peppermint as an Anæsthetic.—Dr. I. J. O'Brien writes (*Druggists' Circular*,): "I became aware of the fact, several years since, in the course of my practice, that the oil of peppermint possessed palliative and anæsthetic properties eminently befitting it as a valuable adjunct to my medicinal cabinet of neuralgic curatives.

"Alternating creosote as a styptic with the oil of peppermint as a sedative,* I have been remarkably successful in the treatment of abscess of the maxillary sinus—alveolar abscess and necrosis of the alveoli—after removing the cause, generally ulcerated teeth or fangs, and as a consequence, necrosed bone.

"In such cases I have always adopted the positive of the antiphlogistic treatment,—dispersed inflammation with the astringent heat of the creosote, and dispelled all painful consequences through the anæsthetical agency of the oil of peppermint."

Chronic Catarrh.—The tincture of aconite in five-drop doses every four hours has cured this troublesome symptom when the ordinary remedies have failed.—*Medical Archives.*

---

*They may be combined.*

Carbolic Acid in Pruritus.—Dr. Binz, of Bonn, has tried this acid in the very obstinate complaint known as pruritus cutaneous. The patient was a man, aged 74, who took gradually from the fifth of a grain to nineteen grains a day. The improvement increased in the same proportion as the doses. Dr. Binz ordered the medicine to be omitted several times, to try whether the favorable changes were the result of chance; but whenever the acid was given up the complaint returned. Dr. Kemmerich tried the acid upon a young soldier affected with pruritus, without success, and gave arsenic with excellent effect.

Treatment of Bursæ in front of the Wrist and Palm of the Hand.—Dr. Frank H. Hamilton, in some remarks on this affection (*Med. Record*, March 1, 1871,) thus speaks of its treatment. A variety of plans have been suggested and practiced with more or less success by surgeons, including incision, excision, injections of iodine, acupuncture, blisters, etc. I am persuaded, however, from my own experience, that no surgical interference is ever proper, except it be to open the sac freely, whenever suppuration takes place. Velpeau relates that in 1822 he saw Prof. Richerand, at the Hopital St. Louis, open a similar tumour in a girl nineteen years old, of a strong constitution. The operation was performed with every necessary precaution, but intolerable pain and violent inflammation ensued, resulting in abscesses, and she was not out of danger until after six weeks of suffering. He adds that he has seen the operation of incision, or of passing a seton through, followed by death in several instances at Hotel Dieu. I have seen a patient succumb to spontaneous inflammation and suppuration of the bursæ; and Mr. Tatum, in Holmes' Surgery, has reported a similar example. Pressure is in some cases serviceable, but generally it will be best to leave the case entirely to Nature, only taking care that it shall not be subjected to injury.

The Treatment of Orchitis.—M. Besnier (*Bull. de Therap.* 1870,) has fouud a simple method of treatment so successful in orchitis of all kinds that he thinks it proper at once to submit it to the consideration of the profession.

The patient is kept at rest, the scrotum is raised, and compresses steeped in a concentrated decoction of the leaves of digitalis are kept constantly applied. The compresses applied may be either lukewarm or cold, and are taken off when they begin to dry. The application should be kept on incessantly. With some folds of cloth under the pelvis and some water-proof around the wet compresses, the application can be carried on with little inconvenience to the patient.—*Dobell's Report on Progress of Med.*

Incontinence of Urine successfully Treated by the Syrup of the Iodide of Iron.—Dr. John Barclay, of Aberdeen, states (Med. Times and Gazette,) that during the past two years and a half, twenty cases of incontinence of urine have been treated by him with the syrup of the iodide of iron alone; and so far as he knows, without a single failure. He gives the details of 11 of these cases. He gave the medicine in doses of from 15 to 30 minims three times a day.

Nitric Acid in Bright's Disease.—Dr. May Figueira, Physician to the Royal Hospital of St. Joseph, at Lisbon, has found great benefit from the use of pure nitric acid mixed with water (as lemonade) in Bright's disease. He has found it superior to tannin, borax, tincture of cantharides, perchloride of iron, etc. He gradually increases the dose to twenty-four and thirty drops four times a day. Milk and raw onions he found most useful in diminishing anasarca and albuminuria.—*Dobell's Report on Progress of Medicine.*

For Skin Affections.—Powdered starch, zinc, lycopodium, etc., are very useful in allaying the heat and itching in acute inflammation of the skin, as erysipelas, shingles, or eruptions attended with moisture. The following is an excellent prescription containing camphor, which is an important adjunct:

| | | | |
|---|---|---|---|
| ℞—Powdered starch, | . . . | dr. vj. | |
| Oxide of zinc, | . . . | dr. iij. | |
| Powdered camphor, | . . . | dr. ss. | |
| Cochineal, | . . . | gr. j. | M. |

To be kept in a stoppered bottle to prevent evaporation.

An Operation for the Cure of Obstructive Dysmenorrhœa.—Prof. T. G. Thomas (*Medical and Surgical Reporter*) in a case of this affection, the obstruction being at the external os which barely admitted a fine probe, removed with a bistoury a small ring of cervical tissue from about the os. He speaks of the operation as devoid of danger.

Internal Complications from Burns.—Microscopical examinations have revealed that the lesions observed in internal organs after local burns or congelations, such as duodenal ulcers, hepatic, pulmonary, and renal hemorrhages, and even cerebral lesions, are the result of embolisms starting from the locally injured parts.

Charcoal in Burns.—A piece of vegetable charcoal laid on a burn at once soothes the pain, says the *Gazette Medicale*, and, if kept applied for an hour, cures it completely.—*Nashville Journal of Medicine and Surgery.*

Abscess of the Mastoid Cells cured by Operation (*Brit. Med. Journ.*, Jan. 28, 1871, p. 88).—A female, aged 40, after suffering for eleven months from abscess of the mastoid cells,—during which period hemicrania and general neuralgia of the fifth pair were prominent symptoms—submitted to the operation of trephining over the mastoid process. The case was under the charge of Dr. F. Buszard, who, in describing his method, says: "I exposed the bone fully by a crucial incision, cutting through the attachment of the sterno-mastoid muscle. A probe was passed through the mastoid foramen, and was found to communicate with the abscess. Pus had been drained off by this opening throughout the history of the case. A small trephine was placed over the foramen, and the outer table of bone removed. At least an ounce of pus escaped. The cavity was then scraped, until the dura mater was reached." The patient made an almost uninterrupted recovery.

Castration for Epileptic Insanity.—Dr. Mackenzie Bacon, in the *Practitioner* for June, cites a case of removal of the testes in a lad who had brought on epilepsy and imbecility by self-abuse. The result was an improvement in every way, including a marked increase of intelligence. Dr. M. thinks the operation would be beneficial to many insane epileptics.—*Pacific Med. Journal.*

Cutaneous Dilatation in Stricture.—Sir Henry Thompson is not an advocate for continuous dilatation in simple stricture, believing that better and safer results are obtained by withdrawing the catheter or bougie immediately after it has entered the bladder (as Luxmoore recommended) than by leaving in the urethra from a few minutes to half an hour, as practiced by some surgeons.—*Dublin Quarterly Journal.*

Carbolic Acid deodorized by the Oil of Lemon.—It is stated (*Eclectlc Med. Jour.*) that "Dr. Yingling, of Huntington, Ind., has found that five drops of oil of lemon will deodorize four ounces of a solution of carbolic acid without any impairment of the medicinal properties of the acid."

Arnica in Pneumonia.—Mr. C. C. Balding recommends strongly (*London Lancet*) the use of arnica (min. x strong tincture every two or three hours) in pneumonia. The pulse should be reduced by it to 60 or 70, and descends at times as low as 40 per minute. The relief is immediate and marked.

To Disguise the Taste of Cod-Liver Oil.—Dr. A. S. Hudson states (*Pacific Medical and Surgical Journal*) that tr. of gum guaiacum half an ounce, and essence of gualtheria one drachm, added to one pint of cod-liver oil, wholly covers the taste of the oil.

